# Genetic Analysis of a Commercial Egg Laying Line Challenged With Newcastle Disease Virus

**DOI:** 10.3389/fgene.2018.00326

**Published:** 2018-08-20

**Authors:** Kaylee Rowland, Anna Wolc, Rodrigo A. Gallardo, Terra Kelly, Huaijun Zhou, Jack C. M. Dekkers, Susan J. Lamont

**Affiliations:** ^1^Department of Animal Science, Iowa State University, Ames, IA, United States; ^2^Hy-Line International, Dallas Center, IA, United States; ^3^School of Veterinary Medicine, University of California, Davis, Davis, CA, United States; ^4^Department of Animal Science, University of California, Davis, Davis, CA, United States

**Keywords:** Newcastle disease virus, GWAS, poultry, disease challenge, genetic parameters, QTL, immune response

## Abstract

In low income countries, chickens play a vital role in daily life. They provide a critical source of protein through egg production and meat. Newcastle disease, caused by avian paramyxovirus type 1, has been ranked as the most devastating disease for scavenging chickens in Africa and Asia. High mortality among flocks infected with velogenic strains leads to a devastating loss of dietary protein and buying power for rural households. Improving the genetic resistance of chickens to Newcastle Disease virus (NDV), in addition to vaccination, is a practical target for improvement of poultry production in low income countries. Because response to NDV has a component of genetic control, it can be influenced through selective breeding. Adding genomic information to a breeding program can increase the amount of genetic progress per generation. In this study, we challenged a commercial egg-laying line with a lentogenic strain of NDV, measured phenotypic responses, collected genotypes, and associated genotypes with phenotypes. Collected phenotypes included viral load at 2 and 6 days post-infection (dpi), antibody levels pre-challenge and 10 dpi, and growth rates pre- and post-challenge. Six suggestive QTL associated with response to NDV and/or growth were identified, including novel and known QTL confirming previously reported associations with related traits. Additionally, previous RNA-seq analysis provided support for several of the genes located in or near the identified QTL. Considering the trend of negative genetic correlation between antibody and Newcastle Disease tolerance (growth under disease) and estimates of moderate to high heritability, we provide evidence that these NDV response traits can be influenced through selective breeding. Producing chickens that perform favorably in challenging environments will ultimately increase the supply of quality protein for human consumption.

## Introduction

In low income countries, chickens play a vital role in daily life. They provide important sources of high quality protein and macro and micronutrients. They are also important for livelihood and gender empowerment, as women are often the beneficiaries of poultry production, which is often not true with larger livestock (Guèye, 2000).

Newcastle disease, caused by avian paramyxovirus type 1, has been ranked as the most devastating disease for scavenging chickens (village chickens, allowed to roam with no to minimal feed provided) in Africa and Asia (Kitalyi, 1998). The more virulent strains of the virus can cause 80% mortality (number of deaths in the flock per infection event) in scavenging flocks (Kitalyi, 1998). High mortality among flocks lead to a devastating loss of dietary protein and buying power for rural households. Prevention of this disease through vaccination is challenging in rural, scavenging production systems. Difficulties arise in ensuring cold chain during transport of vaccines, inadequate vaccination programs, and high costs of administering booster vaccinations (Mayers et al., 2017). Improving the genetic resistance of chickens to NDV is a practical target for improvement of poultry production in low income countries.

Selective breeding has a demonstrated history of success in poultry production (Havenstein et al., 1994) and can be used to modulate many traits of chickens. Several reports have demonstrated genetic differences in response to NDV (Cole and Hutt, 1961; Gordon et al., 1970; Peleg et al., 1976; Soller et al., 1981; Pitcovski et al., 1987). Because response to NDV has a component of genetic control, it can be influenced through selective breeding. Adding genomic information to a breeding program can increase the amount of genetic progress per generation (Fulton, 2012). Recently, there has been a gap in the knowledge accumulation and study of NDV. However, the threat of NDV continues, as demonstrated by the 2018 outbreaks of virulent NDV in California. In this study, we challenged a commercial egg-laying line with a lentogenic (lowly virulent) strain of NDV, measured phenotypic responses, collected genotypes, and associated genotypes with phenotypes. We identified genomic regions associated with response to NDV and/or growth. A selective breeding program can be implemented, e.g., utilizing genomic information identified in this study, to produce chickens that perform favorably in challenging environments and ultimately increase the supply of quality protein for human consumption.

## Materials and Methods

### Animals and Husbandry

The Iowa State University Institutional Animal Care and Use Committee approved all animal procedures and care in this study (log #1-13-7490-G). Pooled semen from 16 sires was used to inseminate 145 dams to produce 3 hatches of 200 mixed-sex chicks (*N* = 600) of a commercial brown egg laying line (Hy-line Brown, Hy-Line International). Birds were provided *ad libitum* access to feed and water throughout the study period. Initially, 23 h of light was provided, which was gradually decreased to 13.5 h of light by day 29. Temperature at chick level on day of placement was 35°C and gradually decreased to 24°C by day 29 and held until completion of the experiment.

### Experimental Design

On day of hatch, chicks were transported to a biosafety level II facility at Iowa State University. For each hatch, chicks were placed into one of three rooms, using pedigree information to distribute half-sibs into different rooms. At 21 days of age (0 dpi), birds were inoculated with 10^8^ of 50% embryonic infectious dose (EID_50_) of live attenuated type B1 LaSota strain NDV in a volume of 200 μL. Virus propagation was detailed previously by Deist et al. (2017b). Virus was administered via a natural, ocular-nasal route. Each eye and nares received 50 μL. Lachrymal fluid samples were collected to quantify viral load at 20, 23, and 27 days of age, hereafter designated as pre-challenge, 2 dpi and 6 dpi, respectively. Blood samples were collected to measure anti-NDV antibody levels on days 20 and 31, hereafter referred to as pre-challenge and 10 dpi, respectively. Body weights were recorded on days 0, 13, 21, 27, and 31 of age. The experimental design was performed across three replicates (3 hatches from the same dams and sires). In each replicate, 180 birds were challenged, 540 in total. The objective of this study is to find genotypic associations with quantitative responses to a viral challenge. Thus, pre-challenge measurements with confirmed null viral load serve as an internal control group.

### Viral Load

To quantify viral load, viral RNA was isolated from lachrymal fluid and quantified via qPCR at three time points: pre-challenge (*n* = 89), 2 dpi (*n* = 468), and 6 dpi (*n* = 470) (**Table 1**). These times were chosen to detect early and maintained viral load (Gallardo, personal communication). Time points also coordinated with related studies (Deist et al., 2017a,b, 2018b; Zhang et al., 2018). Production of lachrymal fluid was induced by placing sodium chloride granules on each eye. The resulting fluid accumulation was collected with a pipette. Viral RNA was isolated from the lachrymal fluid using a MagMAX-96 viral RNA isolation kit (Life Technologies, Carlsbad, CA, United States). Isolated RNA was quantified using an LSI VetMAX NDV real-time PCR kit (Life Technologies, Carlsbad, CA, United States) targeted to the matrix protein (M) gene of NDV. Viral RNA was isolated once per sample and quantified via qPCR in duplicate. Mean viral RNA copy number was calculated per sample and log transformed. To test the difference between time points, least squares means were calculated and Student’s *t*-test were performed in JMP (SAS Institute, Inc., Cary, NC, United States). In calculating least squares means, effects included qPCR plate, day, room nested within replicate, and sex.

**Table 1:** Descriptive statistics of phenotypes and estimates (SE) of variance components (proportions of phenotypic variance).

Trait	*N*^3^	Mean^4^	*SD*^5^	Heritability	Dam	Residual
Viral load 2 dpi^1,2^	468	5.10	0.60	0.32 (0.1)	–	0.68 (0.02)
Viral load 6 dpi^1,2^	470	3.72	0.91	0.18 (0.1)	–	0.82 (0.07)
Dam antibody^1^	139	0.53	0.22	–	–	–
Antibody pre-challenge^1,2^	453	-0.84	0.60	0.26 (0.09)	0.51 (0.01)	0.23 (0.00)
Antibody 10 dpi^1,2^	448	-0.06	0.26	0.24 (0.09)	–	0.76 (0.01)
Growth rate pre-challenge^2^	473	10.4	1.20	0.46 (0.11)	0.08 (0.04)	0.47 (0.06)
Growth rate post-challenge^2^	470	15.4	2.76	0.21 (0.09)	–	0.79 (0.3)



*^1^Phenotypes log_10_ transformed. ^2^Outliers (> 3SD <) removed. ^3^Number of phenotypic records in the association analysis. ^4^Arithmetic mean. ^5^Standard deviation.*

### Antibody

Anti-NDV antibody levels in sera were quantified pre-challenge (*n* = 453) and at 10 dpi (*n* = 448) using an IDEXX NDV ELISA for chickens (IDEXX Laboratories, Inc., Westbrook, ME, United States) (**Table 1**). This is the time needed (10 dpi) to generate an acquired immune response (production of specific antibodies). This time also coordinated with related studies (Deist et al., 2017a,b, 2018b; Zhang et al., 2018). Each sample was quantified in duplicate and the average sample:positive (S/P) absorbance ratio was calculated per manufacturer’s instructions. To test the difference between time points, a standard least squares effect leverage report and Student’s *t*-test were performed in JMP (SAS Institute, Inc., Cary, NC, United States). Effects included day, room nested within replicate, and sex. Antibody levels were also quantified, just prior to the second hatch, on dams (*n* = 139), which had received multiple vaccines against NDV over their lifetime, using the same assay, except plasma was used instead of serum.

### Growth Rate

Body weights were recorded in grams on days 0, 13, 21 (0 dpi), 27 (6 dpi), and 31 (10 dpi). Pre-challenge growth rate (*n* = 473) was calculated as grams per day between days 0 and 21. Post-challenge growth rate (*n* = 470) was calculated as grams per day between days 21 and 31.

### Genotyping

Whole blood was collected on Whatman FTA cards (Sigma-Aldrich, St. Louis, MO, United States) from all chicks pre-challenge. Genomic DNA was isolated from FTA card punches, dried, and shipped to GeneSeek, Neogen Genomics (Lincoln, NE, United States). DNA was genotyped for 600,000 SNPs using the Axiom Chicken Genotyping Array (Kranis et al., 2013) (Thermo Fisher Scientific, Inc., Waltham, MA, United States). Axiom Chicken Genotyping Array annotation files, release 35, were based on galGal genome version 5.0 (Thermo Fisher Scientific). Quality filtering of genotype data included call rate ≥95 and minor allele frequency ≥0.01. Other filtering metrics (Nclus, FLD, HomRO, HomFLD, HetSO, ConversionType, BB.varX, BB.varY, AB.varX, AB.varY, AA.varX) and requirements are listed in **Table 2**. These metrics are described in the Axiom Analysis Suite User Guide obtained from Thermo Fisher Scientific (Applied Biosystems, 2017).

**Table 2:** Genotype quality metrics provided by Affymetrix and requirements that were used in quality control filtering.

Affymetrix genotype metric	Requirement	Brief description of metric^1^
Nclus	≠ 1	Number of genotype clusters
Call rate	≥95	% of samples with a genotype call other than “No Call”
MinorAlleleFrequency	≥0.01	min(P_A_, P_B_)
FLD	≥3.5	Measure of the cluster quality of a probeset
HomRO	≥-0.988	Distance to zero in the Contrast dimension (X position) from the center of the homozygous cluster that is closest to zero
HomFLD	≥10	Measure of the cluster quality of a probeset for the homozygous genotype clusters
HetSO	≥-0.21	Measures how far the heterozygous cluster center sits above the homozygous cluster centers in the Size dimension (Y)
ConversionType	≠ OTV	Probeset classification
BB.varX	≤0.85	Contrast (X position) variance for BB cluster
BB.varY	≤0.7	Size (Y position) variance for BB cluster
AB.varX	≤0.75	Contrast (X position) variance for AB cluster
AB.varY	≤0.75	Size (Y position) variance for AB cluster
AA.varX	≤0.79	Contrast (X position) variance for AA cluster



*^1^For detailed description of metrics see Axiom Analysis Suite User Guide (Applied Biosystems, 2017).*

### Genetic Parameters

Variance components and heritabilities were estimated in ASReml 4 (Gilmour et al., 2015) using the following univariate animal model:

(1) $$Y_{\mathrm{ijk}}{=µ\;+\;S}_{i}{\;+\;\mathrm{RR}}_{j}{\;+\;A}_{k}{\;+\;e}_{\mathrm{ijk}}$$

where Y is the dependent variable of phenotype (viral load 2 and 6 dpi, antibody pre-challenge and 10 dpi, growth rate pre and post-challenge). Sex (S) and a combined variable of room and replicate (RR) were fitted as fixed effects. Random effects included animal genetic effects (A) with a genomic relationship matrix (GRM) computed from SNP genotypes following the procedure described by VanRaden (2008), and residuals (e). For viral load at 2 and 6 dpi, qPCR plate was also added as a fixed effect, and for antibody pre-challenge, antibody level of the dam was added as a covariate. The random effect of dam was included for pre-challenge measurements of growth rate and antibody. Phenotypic variance was obtained by summing estimates of variance due to animal, residual, and dam (where applicable). Heritability was calculated as a ratio of the estimates of animal to phenotypic variance.

### Association Analysis

Association analyses were performed using the R package GenABEL (Aulchenko, 2015), using a hierarchical generalized linear model (Rönnegård et al., 2010) with the same fixed effects as described for estimation of genetic parameters. The “*polygenic_hglm*” function was used to fit a polygenic model, with a GRM that was created by GenABEL using the *ibs()* function with the *weight = “no”* option. The “*mmscore*” function, which is designed to test for association between a trait and genetic polymorphism in samples of related individuals, was used with residuals from *polygenic_hglm* analysis. The *mmscore* function uses the formula

(2) $$\frac{{\left((G-E[G])V^{-1}\mathrm{residualY}\right)}^{2}}{{(G-E[G])V}^{-1}(G-E[G])}$$

where G is the vector of SNP genotypes, E[G] is a vector of mean genotypic values, V^-1^ is the inverse of variance-covariance matrix, and residualY are residuals from the trait analysis with *polygenic_hglm*. Together score test for association) method implemented by Chen and Abecasis (2007).

### Multiple Test Correction

Genotypes were divided into chromosomes and then further divided into chromosomal segments containing a number of SNPs equal to half the number of animals as described by Waide et al. (2017). The number of independent tests was determined as the sum number of principle components that accounted for 95% of variance between genotypes for each segment (Σ*n*). The number of independent tests was used in a Bonferroni correction to determine 20% suggestive genome-wide thresholds as 0.2/Σ*n*.

## Results

### Viral Load

**FIGURE 1:**
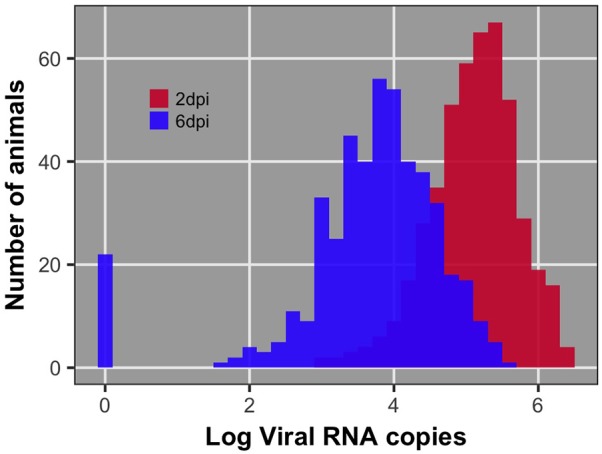
Distribution of viral load at 2 and 6 days post-infection (dpi) after log10 transformation. The bar at 0 for 6 dpi reflects the 22 individuals that had no detectable viral RNA at 6 dpi. These individuals were recorded as having 0 viral RNA copies.

**FIGURE 2:**
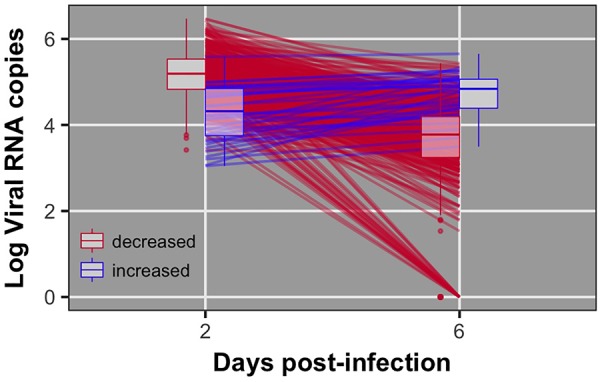
Individual data and box plots for viral load at 2 and 6 dpi. Red lines indicate birds that decreased viral load from 2 to 6 dpi. Blue lines indicate birds that exhibited increased viral load from 2 to 6 dpi. 22 birds did not have detectable virus at 6 dpi.

Pre-challenge samples had no measurable virus copies, as expected (data not shown). Distributions of viral load 2 and 6 dpi are shown in **Figure 1**. Viral load was significantly different between 2 and 6 dpi (*P* < 0.0001). Viral load was greater at 2 than 6 dpi for all but 38 birds (8%) (**Figure 2**). By 6 dpi, 22 birds fell below our limit of detection for measurable viral RNA, indicative of viral clearance.

### Antibody

**FIGURE 3:**
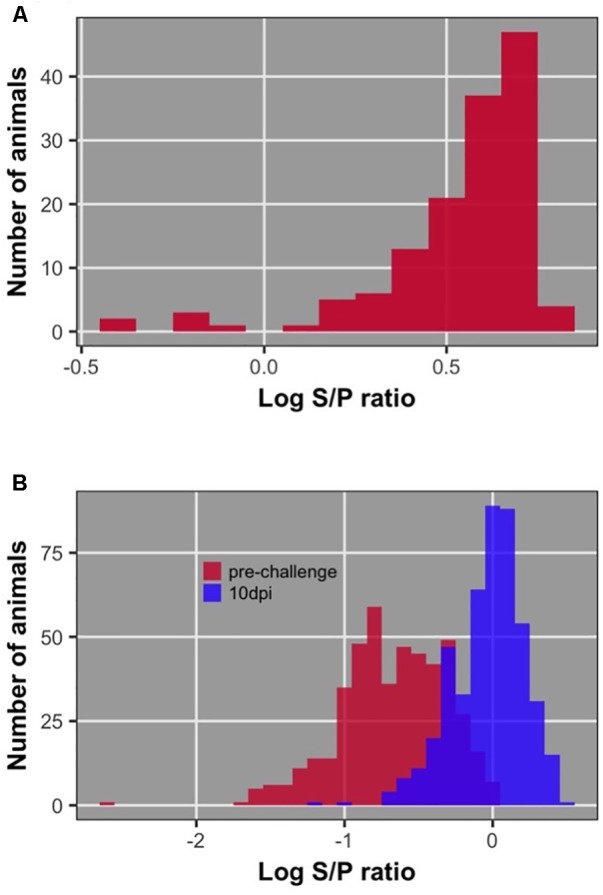
Distribution of antibody level after log10 transformation; **(A)** of dams; **(B)** of offspring pre-challenge and 10 dpi.

**FIGURE 4:**
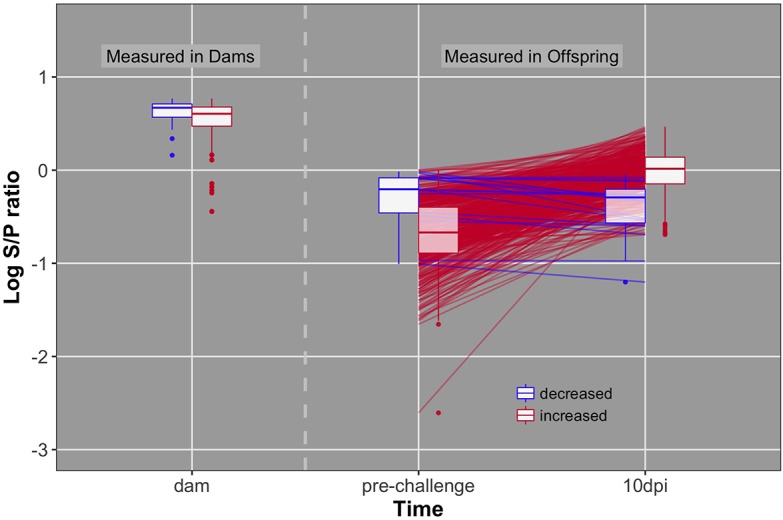
Individual data and box plots for antibody pre-challenge and 10 dpi. Red lines and boxplots indicate animals that increase antibody levels in response to challenge (pre to 10 dpi). Blue lines and boxplots indicate animals that do not increase antibody levels in response to challenge. Dams included in blue and red boxplots produced offspring that decreased and increased antibody levels, respectively.

Distributions of dam, chick pre-challenge, and chick 10 dpi antibody are shown in **Figure 3**. Pre-challenge anti-NDV antibody levels were measurable but significantly lower than antibody levels at 10 dpi (*P* < 0.0001) for all but 19 birds (4%) (**Figure 4**). These 19 birds were excluded from association analysis for both antibody time points. Antibody levels measured in dams were significantly higher than either pre-challenge or at 10 dpi in their chicks.

### Growth Rate

**FIGURE 5:**
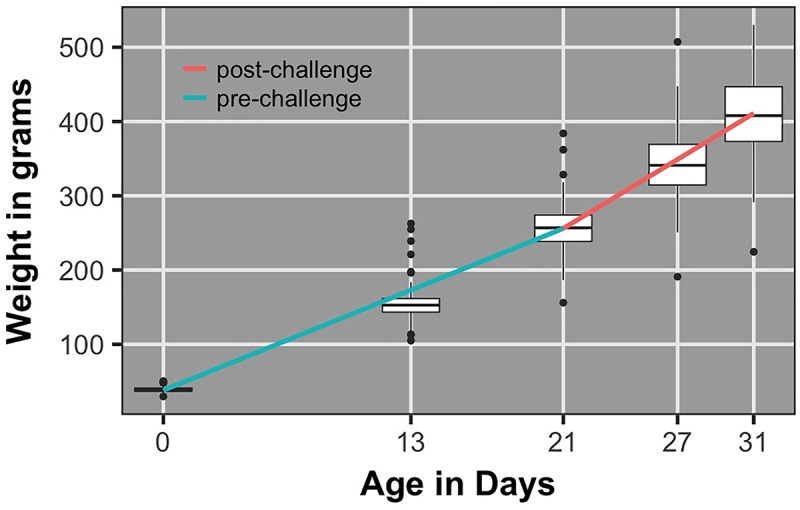
Box plots of body weights and regression lines for population average growth rate pre- and post-challenge.

**Figure 5** shows the population average growth rate pre- and post-challenge and corresponding body weight box plots. Growth rate post-challenge was significantly greater (*P* < 0.0001) than growth rate pre-challenge.

### Phenotypic Correlations

Viral load at 2 and 6 dpi were positively correlated (**Table 3**). Pre-challenge antibody level was negatively correlated with both pre and post-challenge growth rate. Post-challenge growth rate was negatively correlated with viral load at 2 dpi and antibody level pre-challenge, but positively correlated with pre-challenge growth rate.

**Table 3:** Estimates (SE) of phenotypic (above diagonal) and genetic (below diagonal) correlations based on bivariate analyses.

Trait	VL 2 dpi	VL 6 dpi	AB pre	AB 10 dpi	GR pre	GR post
Viral load 2 dpi	–	0.18 (0.06)	0.04 (0.05)	0.01 (0.05)	-0.05 (0.06)	-0.16 (0.05)
Viral load 6 dpi	0.74 (0.21)	–	-0.03 (0.05)	-0.06 (0.05)	-0.02 (0.06)	-0.05 (0.05)
Antibody pre-challenge	0.12 (0.28)	-0.25 (0.34)	–	0.06 (0.05)	-0.22 (0.05)	-0.12 (0.05)
Antibody 10 dpi	0.13 (0.31)	-0.39 (0.33)	0.33 (0.34)	–	-0.05 (0.05)	-0.07 (0.05)
Growth rate pre-challenge	-0.11 (0.22)	0.23 (0.32)	-0.72 (0.19)	-0.42 (0.29)	–	0.58 (0.04)
Growth rate post-challenge	-0.30 (0.26)	0.21 (0.37)	-0.61 (0.28)	-0.45 (0.33)	0.72 (0.14)	–


### Heritabilities

Heritabilities estimated using AsReml4 were moderate (0.18 to 0.32) for viral load (**Table 1**). Estimates of heritability for pre- and post-challenge antibody levels were similar, 0.26 and 0.24, respectively. Estimates of heritability for Pre- and post-challenge growth rate were moderate, 0.46 and 0.21, respectively.

### Genetic Correlations

The estimate of the genetic correlation between viral load at 2 and 6 dpi was high, 0.74 ± 0.21 (**Table 3**). Viral load at 6 dpi and antibody at 10 dpi were negatively correlated (-0.39 ± 0.33); birds with more antibodies had lower viral load. Most pathogen challenge-related traits, with the exception of viral load 6 dpi, were negatively correlated with growth rate pre- and post-challenge (-0.30 to -0.72). The two measures of growth rate had a high positive genetic correlation of 0.72. Standard errors for genetic correlation estimates were moderate, leading some estimates to not differ from 0.

### Alternative Phenotypes

Several alternative phenotypes generated by combination and/or manipulation of individual phenotypes collected in this study were explored: viral load and antibody change over time (difference between time points), viral load clearance (difference between time points divided by 2 dpi level), regression of viral load and antibody measurements over time. However, none were more heritable than the individual phenotypes and most did not have heritability different from 0. Thus, they were not included further in this study.

### Association Analysis

After quality control, 476 animals and 340,527 SNPs remained for association analysis. Principle component analysis determined that 44,364 components accounted for 95% of variance between SNPs. Using 44,364 as the number of independent tests and applying Bonferroni correction, the 20% genome-wide significance threshold was 4.508 × 10^-6^ and used to declare suggestive associations.

**FIGURE 6:**
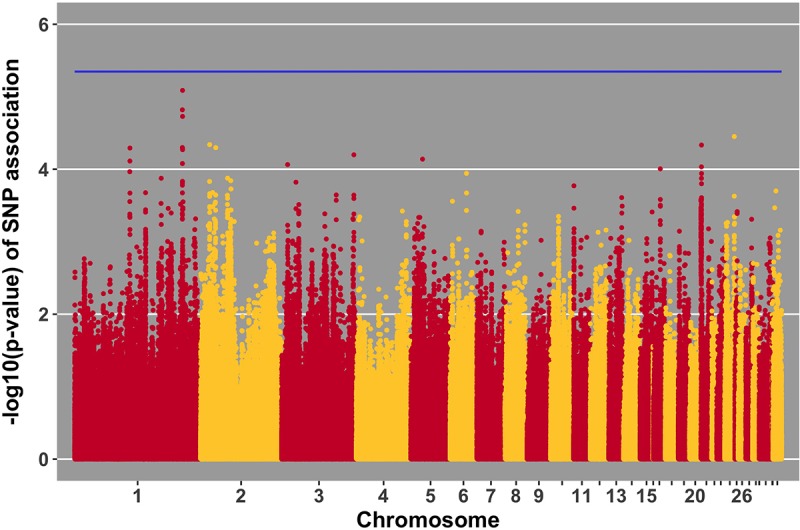
Manhattan plot for viral load at 2 dpi; 0 SNPs reached the 20% genome-wide threshold indicated by the blue line.

**FIGURE 7:**
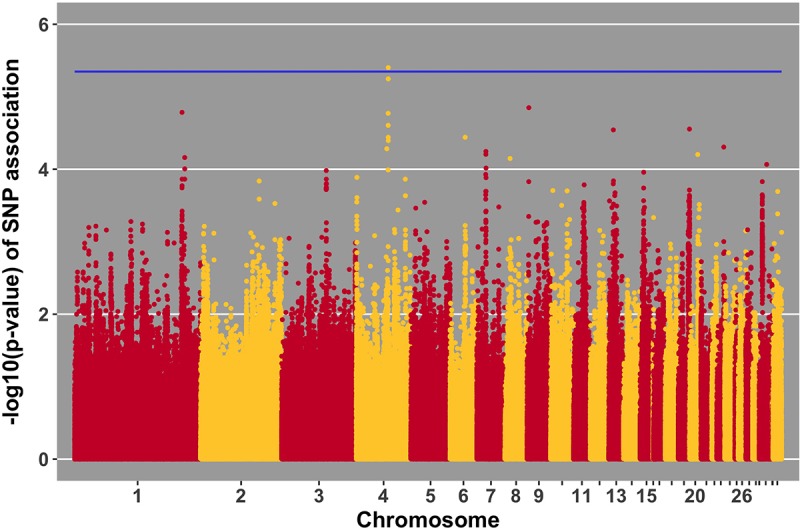
Manhattan plot for viral load at 6 dpi; 1 SNP reached the 20% genome-wide threshold indicated by the blue line.

**FIGURE 8:**
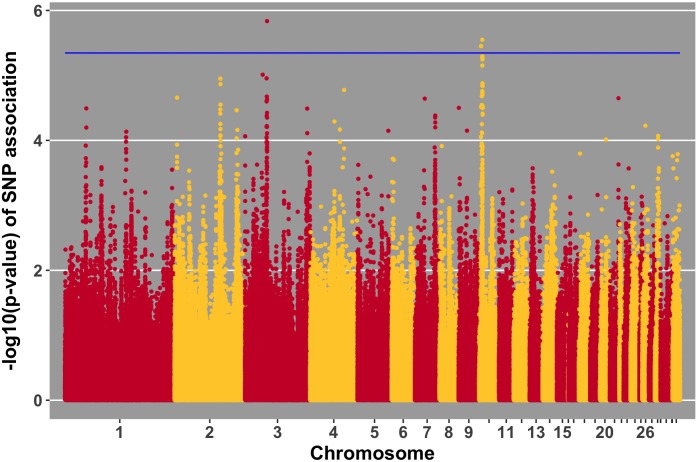
Manhattan plot for antibody level pre-challenge; 3 SNPs reached the 20% genome-wide threshold indicated by the blue line.

**FIGURE 9:**
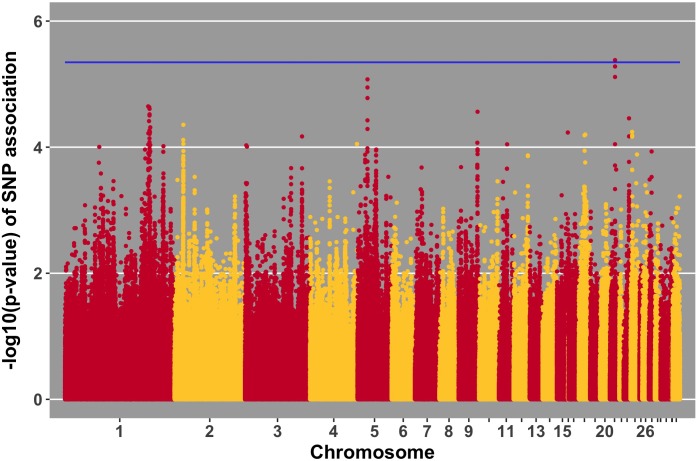
Manhattan plot for antibody 10 dpi, 1 SNP reaches the 20% genome-wide threshold indicated by the blue line.

**FIGURE 10:**
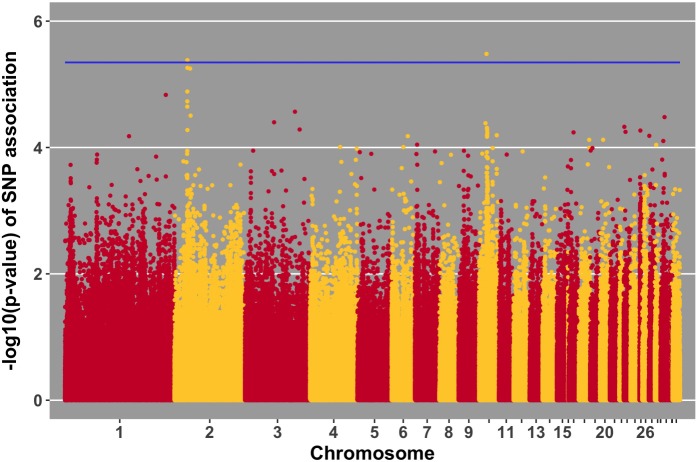
Manhattan plot for growth rate pre-challenge; 2 SNPs reached the 20% genome-wide threshold indicated by the blue line.

**FIGURE 11:**
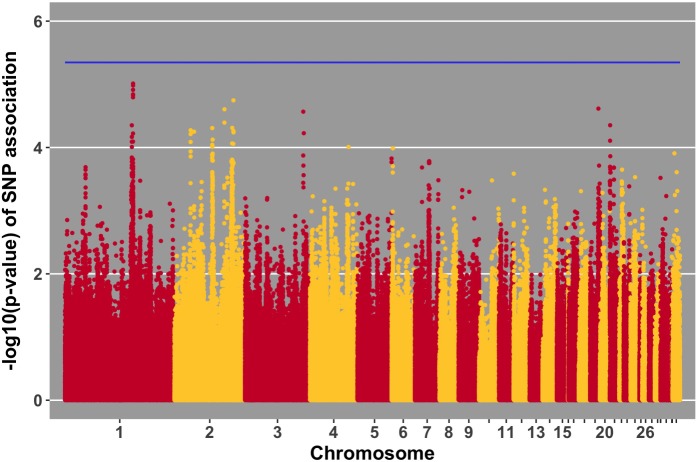
Manhattan plot for growth rate post-challenge, 0 SNPs reach the 20% genome-wide threshold indicated by the blue line.

Manhattan plots for viral load at 2 and 6 dpi are in **Figures 6, 7**, respectively. One SNP on chromosome 4 was associated with viral load at 6 dpi, while none were associated with viral load 2 dpi (**Table 4**). Association analysis results are reported for antibody pre-challenge and 10 dpi, excluding the 19 birds that did not increase antibody in response to NDV challenge. Manhattan plots for antibody level pre-challenge and at 10 dpi are in **Figures 8, 9**, respectively. Three SNPs were associated with antibody level pre-challenge, while one SNP was associated with antibody level at 10 dpi (**Table 4**). Two SNPs were associated with growth rate pre-challenge, while none were associated with growth rate post-challenge (**Figures 10, 11** and **Table 4**).

**Table 4:** Single nucleotide polymorphisms associated with traits, positional candidate genes, and previously reported QTL.

Trait	SNP	Position^1^	*P*-value	Positional Candidate Genes and Location^2^	Previous relevant QTL associations
Antibody pre-challenge	AX-76468260	3:38180614	1.31E-06	*B3GALNT2*, intron	Antibody titer to LPS antigen^5^
				*GPR137B*, upstream 213694	
				*NTPCR*, downstream 954041	
	AX-75608938^4^	10:4095431	2.57E-06	*LACTB*, intron	Antibody titer to LTA antigen^5^
	AX-75605132	10:2826316	3.21E-06	*LINGO1*, downstream 58378	
				*HMG20A*, downstream 66323	
Antibody 10 dpi	AX-76244799	21:3996542	4.00E-06	*TARDBP*, upstream 65501	None related to pathogen response
				*APITD1*, downstream 267277	
				*CASZ1*, upstream 51894	
Viral Load 6 dpi	AX-76683655	4:53179704	4.07E-06	*FAT4*, upstream 191754	Marek’s disease-related traits^6^
				*ANKRD50*, upstream 38776	
				*SPRY1*, downstream 534821	
				*SULT1B1*, upstream 1040403	
Growth rate pre-challenge	AX-75623995	10:8913444	3.29E-06	*MAPK6*, synon^3^	Carcass weight^7^
	AX-80773317^4^	2:27242600	4.15E-06	Novel lincRNA, downstream 384292	Body weight^8,9,10^
				Novel lincRNA, downstream 82294	
				*SCIN*, downstream 399726	
				*ER81*, downstream 85619	


*^1^Chromosome:base pair. ^2^Location of positional candidate gene (bp from the SNP). ^3^SNP is within a coding sequence but does not result in a residue change. ^4^SNP is fixed for alternate alleles in Fayoumi and Leghorn inbred lines. ^5^Siwek et al. (2006). ^6^Xu et al. (1998). ^7^Nassar et al. (2012). ^8^Siwek et al. (2004). ^9^Tatsuda and Fujinaka (2001). ^10^Uemoto et al. (2009).*

## Discussion

### Genetic Parameters

Heritabilities for all traits were estimated to be moderate to high, ranging from 0.18 for viral load at 6 dpi to 0.46 for growth rate pre-challenge. Our heritability estimates for antibody levels at 10 dpi are in line with those reported by (Lwelamira et al., 2009) in two Tanzanian chicken ecotypes measured just prior to and 2 weeks post-vaccination (0.27 and 0.29). Peleg et al. (1976) estimated heritability of antibody response to attenuated NDV at 12 dpi to be 0.31 based on the sire variance components. To our knowledge, ours is the first report of heritability for viral load of NDV and growth rate in layer-type birds. The moderate to high heritabilities estimated in this study indicate that all investigated traits can be influenced by selective breeding. Therefore, the means for these traits can be changed over generations.

Negative genetic correlations between pathogen response traits and growth rates indicate that selection for decreased viral load at 2 dpi and for decreased antibody levels is expected to increase pre- and post-challenge growth rate. Many studies have found immune response traits and production/growth traits under challenge to be negatively genetically correlated (Gross et al., 2002; Lwelamira et al., 2009; Hess et al., 2016). Given this information, we can speculate that higher antibody levels, which are often viewed as favorable, may be unfavorable when the desired outcome is to increase disease tolerance. Tolerance is defined as the ability of a host to limit the negative impact of infection (viral in this case) on performance (Bishop, 2012). Tolerance is a good goal for NDV in low income countries, where the virus is relatively ubiquitous and the majority of animals will be infected by the virus at some point in their life. Furthermore, it has been suggested that host tolerance places less pressure on the virus to evolve (Råberg et al., 2009). It must be recognized, however, that standard errors for genetic correlation estimates were moderate, leading some estimates to not differ from 0. A larger sample size will be needed to determine the true significance of genetic correlations.

The genetic correlation between pre- and post-challenge growth and viral load at 6 dpi was positive, which does not fit the previously mentioned negative trend between pathogen response traits and growth rates, although SE estimates were large. The resource allocation argument may provide an explanation in this case (Gross et al., 2002; Rauw, 2012). Birds that have higher viral load at 6 dpi also have higher pre- and post-challenge growth rates because they use more of their available resources to grow as opposed to clearing the virus.

### Viral Load

The 38 birds (8%) that increased viral load from 2 to 6 dpi represent a different kinetic profile of viral clearance than the rest of the population. Although these 38 birds exhibited a different pattern of viral clearance, there was no evidence for lack of infection or interference of response to challenge. There is no evidence that these birds were less challenged, as all 38 had measurable viral load at 2 dpi, indicating they were infected with NDV. Furthermore, none of the 38 birds were half- or full-sibs to the 19 birds that did not produce antibody in response to challenge. Viral load heritability estimates were not increased by excluding these 38 birds. Therefore, these birds were not excluded from any analyses.

No SNPs reached the suggestive threshold for viral load at 2 dpi, while one SNP reached that threshold for viral load at 6 dpi. For this SNP, located on chromosome 4 at 53 Mb, four genes were located within 1 Mb. This QTL was previously identified in association with Marek’s disease-related traits (Xu et al., 1998). The closest gene, *ANKRD50*, was previously found to be down-regulated in tracheal epithelial cells of an inbred research line of Fayoumi chickens at 2 dpi with NDV compared to non-infected birds (Deist et al., 2017b). Chickens from Fayoumi and Leghorn inbred lines were used in a companion study that had the same experimental design, used the same virus, and measured the same phenotypes as the current study. Deist analyzed transcriptome responses of trachea, lung, and Harderian gland to NDV challenge (Deist et al., 2017a,b, 2018b). Zhang reported transcriptomic changes in the spleen (Zhang et al., 2018). The Fayoumi and Leghorn lines are highly inbred (Fleming et al., 2016) and their responses to various pathogens, including velogenic NDV (Lakshmanan et al., 1996; Cheeseman et al., 2007; Kim et al., 2008; Wang et al., 2014, Deist, 2018a), demonstrate the Fayoumi and Leghorn lines to represent relatively resistant and susceptible genetic research models, respectively. *ANKRD50* functions in endosome to plasma membrane transport (Kvainickas et al., 2017). This is the first reported association of *ANKRD50* with viral infection.

### Antibody

It was not expected to have detectable pre-challenge antibody at 20 days of age, because many reports have demonstrated clearance of maternally transferred antibody by this age (Rose and Orlans, 1981; Liu and Higgins, 1990; Grindstaff et al., 2003; Hamal et al., 2006). However, the dams of challenged chicks were ‘hyperimmunized,’ as they had received 5 immunizations for NDV prior to production of the chicks used in this study. Thus, we believe that the passive maternal antibody still circulating at 20 days of age may have interfered with the response to NDV challenge, specifically in the 19 chicks that did not increase level of antibody between pre-challenge and 10 dpi. Maternal antibody interference with vaccine response is a known phenomenon (Richey and Schmittle, 1962; Eidson et al., 1976). Because these 19 chicks were likely unable to respond to the vaccine appropriately because of passive antibody interference, we conducted analyses both with and without these chicks included. Heritability of pre-challenge antibody increased from 0.20 to 0.26 with exclusion of these birds. The same trend of increasing heritability was seen for antibody 10 dpi, from 0.19 to 0.24. Three suggestive QTL were found when excluding these 19 birds, while only two of the three were found when using the full dataset. These analyses provide evidence that passive antibody interference caused “noise” in the antibody response data; therefore these 19 birds were excluded from the association analysis for antibody pre-challenge and at 10 dpi. We expect that dams in low income countries would also have relatively high amounts of anti-NDV antibodies due to high environmental levels of NDV and repeated exposure to the virus.

Pre-challenge antibody did not differ significantly between the three replicates suggesting that maternal antibody transfer level did not differ significantly due to the time between the three hatches. Dam antibodies were measured from plasma, while chick antibodies were measured from serum. Previous studies have shown that antibody measured in plasma and serum are highly correlated (Cherpes et al., 2003; Siev et al., 2011), suggesting the validity of comparing levels of antibody between dams’ plasma and chicks’ serum in the current study.

Three SNPs, in two QTL, were suggestively associated with antibody level pre-challenge. The strongest association was on chromosome 3 at 38.2 Mb. This SNP was within the intron of *B3GALNT2*. *B3GALNT2* was previously found to be more highly expressed in the Harderian gland of Fayoumis compared to Leghorns at 2 days post-NDV inoculation (Deist et al., 2018b). *B3GALNT2* functions in protein glycosylation (Stevens et al., 2013). We present a novel association of *B3GALNT2* with viral infection. One gene, *GPR137B*, was near the SNP, 213,694 bp upstream. *GPR137B* is a lysosomal integral membrane protein predicted to function in signal transduction (Gao et al., 2012). The QTL on chromosome 3 for antibody level pre-challenge, was previously associated with antibody titer to LPS antigen (Siwek et al., 2006).

The second QTL for antibody pre-challenge on chromosome 10 contained two SNPs. The strongest SNP within the chromosome 10 QTL was within the intron of the *LACTB* gene. This SNP was fixed for alternate alleles in the Fayoumi and Leghorn lines, evaluated by 600k Axiom Chicken Genotyping Array data from 10 birds per line. *LACTB* promotes intra-mitochondrial membrane organization through polymerization (Polianskyte et al., 2009). This is the first identified association of *LACTB* with antibody production.

The second SNP within the chromosome 10 QTL was near two genes, *LINGO1* and *HMG20A*. *LINGO1* was previously found to be down-regulated in the lung of Fayoumi chickens at 2 dpi with NDV, compared to non-infected birds (Deist et al., 2017a). *LINGO1* was also less expressed in the Harderian gland of Fayoumis compared to Leghorns at 2 days after challenge with NDV (Deist et al., 2018b). When comparing the expression in the lung of non-challenged birds, Fayoumi chickens expressed more *LINGO1* than Leghorns (Deist et al., 2017a). *LINGO1* is a transmembrane protein functioning in signal transduction (Mi et al., 2004). *HMG20A* exhibited more expression in the lung of non-challenged Leghorn chickens compared to Fayoumi chickens (Deist et al., 2017a). In tracheal epithelial cells at 2 and 10 days post-NDV infection, Leghorns expressed more *HMG20A* than Fayoumis (Deist et al., 2017b). *HMG20A* has been shown to bind to viral DNA *in vitro* (Hsiao et al., 2006). The second antibody pre-challenge QTL on chromosome 10 was previously associated with antibody titer to LTA antigen (Siwek et al., 2006).

Antibody level at 10 dpi was associated with one SNP, located at 3.9 Mb on chromosome 21. Three genes were nearby, *TARDBP, APITD1*, and *CASZ1*. *CASZ1* was down-regulated 2 days post-NDV challenge in tracheal epithelial cells of Leghorn chickens compared to non-challenged birds (Deist et al., 2017b). *TARDBP* functions in negative regulation by host of viral transcription (GO biological process) and was previously implicated as part of the influenza-host interactome using human and mammalian cell lines *in vitro* (Heaton et al., 2016).

### Growth Rate

Compared to the management guide for the Hy-Line Brown commercial layers, our birds had higher body weights across all weeks partially due to the inclusion of male chicks in our experimental population (Hy-Line International, 2016). However, the growth rate trajectories between our birds and the management guide are roughly parallel, suggesting we are not seeing a large depression due to challenge.

Growth rate pre-challenge was associated with two SNPs on chromosomes 10 and 2. The SNP on chromosome 10 was within the *MAPK6* gene, which functions in phosphorylation. This QTL co-localizes with a previous association for carcass weight (Nassar et al., 2012).

The *ER81* gene, near the SNP for pre-challenge growth rate on chromosome 2, functions in transcription regulation. This QTL has been previously been identified to be associated with body weight in three independent populations (Tatsuda and Fujinaka, 2001; Siwek et al., 2004; Uemoto et al., 2009).

### Support of Expression Studies for Suggestive SNP Associations

Incorporating previous gene expression data can improve the value of GWAS data, especially when significant expression data coincides with suggestive (near-significant) SNPs (Cheng et al., 2013). The SNP on chromosome 5 with the lowest *p*-value of association with viral load at 2 dpi (**Figure 6**), is within a gene (*PAMR1*) that was previously found to be differentially expressed in tracheal epithelial cells at 2 and 6 days post-NDV infection (Deist et al., 2017b). At both time points, Leghorn chickens expressed higher levels of *PAMR1* compared to Fayoumis. The Leghorn chickens in the referenced study had significantly more viral genome transcripts in the trachea at 2 dpi. Perhaps the difference in viral load between the two lines is due in part to expression differences in this gene and provide support for the near significant GWAS results.

We identified a suggestive QTL on chromosome 4 at 53 Mb for viral load at 6 dpi (**Figure 7**). Several SNPs in the location of the QTL fell just below the threshold. One of these SNPs (*p*-value of association 5.22 × 10^-5^) is within the *ADAMTS3* gene. In samples from the birds utilized in this GWAS study, the *ADAMTS3* gene was shown to be down-regulated in the spleen 6 days after NDV challenge (Zhang et al., 2018). Integration of this information provides further evidence for the existence of the identified QTL for viral load at 6 dpi on chromosome 4.

The *OFD1* gene encompasses three SNPs within the near-significant QTL on chromosome 1 for growth rate post-challenge (**Figure 11**). *OFD1* functions in primary cilium organization and assembly (Ferrante et al., 2006). This gene was shown to exhibit lower expression in the Harderian gland of Leghorns compared to Fayoumis, 2 days after NDV challenge (Deist et al., 2018b). Perhaps the differential expression of *OFD1* contributes to the susceptible/resistant phenotypes of the Leghorn/Fayoumi lines. Overall, *OFD1* may play a role in NDV tolerance – performance (growth) under challenge.

## Conclusion

Six suggestive QTL associated with response to NDV and/or growth were identified. Some were novel and others confirmed previously reported associations with related traits. Additionally, previous RNA-seq analysis provided support for several of the genes located in or near the QTL of the current study. Considering the trend of negative genetic correlation between antibody and Newcastle Disease tolerance (growth under disease) and estimates of moderate to high heritability, we provide evidence that these NDV response traits can be influenced through selective breeding. This information can inform breeding decisions for the production of chickens that will be raised in NDV endemic areas once more knowledge of the relationship of antibody and viral load with mortality is obtained. Producing chickens that perform favorably in challenging environments will ultimately increase the supply of quality protein for human consumption.

## Availability of Data and Materials

The data that support the findings of this study are available from Hy-Line International but restrictions apply to the availability of these data, which were used under license for the current study, and thus are not publicly available. However, data are available from the authors upon reasonable request to SL and with permission of Hy-Line International.

## Author Contributions

HZ, RG, JD, and SL designed the study. KR, AW, JD, and SL performed the animal experiments and collected phenotypes. KR performed the lab work related to quantifying phenotypes and DNA extraction. KR performed the data analysis with inputs from AW, JD, HZ, and SL. KR wrote the initial draft of the manuscript. KR, AW, RG, TK, HZ, JD, and SL provided critical revision. All authors read and approved the final manuscript.

## Conflict of Interest Statement

Hy-Line International made the in-kind contribution of the animals studied. AW is employed by Hy-Line International and Iowa State University. AW helped with the animal experiments and phenotype collections and provided input on data analysis. Hy-Line International is interested in the outcome of this experiment in regards to their commercial product, but this had no influence on the outcomes of the experiment or this manuscript. The remaining authors declare that the research was conducted in the absence of any commercial or financial relationships that could be construed as a potential conflict of interest.

## Abbreviations

dpi: days post-infection
EID_50_: 50% embryo infectious dose
ELISA: enzyme-linked immunosorbent assay
GRM: genomic relationship matrix
Mb: mega base
NDV: Newcastle disease virus
QTL: quantitative trait loci
SE: standard error
SNP: single nucleotide polymorphism
